# SOCIAL RELATIONSHIPS AND DUAL FUNCTIONALITY IN LATER LIFE

**DOI:** 10.1093/geroni/igad104.1870

**Published:** 2023-12-21

**Authors:** Patricia Thomas, Madison Sauerteig-Rolston, Shawn Bauldry, Samuel Nemeth, Kenneth Ferraro

**Affiliations:** Purdue University--West Lafayette, West Lafayette, Indiana, United States; Purdue University, West Lafayette, Indiana, United States; Purdue University, West Lafayette, Indiana, United States; Purdue University, West Lafayette, Indiana, United States; Purdue University, West Lafayette, Indiana, United States

## Abstract

Longer lives are often accompanied by greater risk of cognitive and physical impairments, but we investigate whether social relationships reduce the risk of those impairments. We examine dual functionality – the absence of both dementia and activities of daily living impairments – and draw upon stress process and social integration theories to examine the impact of positive and negative dimensions of relationship quality with friends and family to better understand how social factors may affect how long people maintain both their physical and cognitive functioning in later life. We use nationally representative panel data from the Health and Retirement Study (2006-2016, N=6,985) and Weibull accelerated failure-time models. Higher strain with family members and with friends was associated with a loss of dual functionality at earlier ages (Time Ratio = .97 [95% CI: .95, .99] and TR = .96 [CI: .94, .98], respectively). Greater social support from family members was beneficial, related to a longer time as dual functional (TR = 1.04, CI: 1.02, 1.06); however, more support from friends was related to loss of dual functionality at earlier ages (TR = .98, CI: .96, .99). Findings reveal the implications of social support and strain in different types of relationships for dual functionality.

